# Expression of *six3* and *otx* in Solenogastres (Mollusca) supports an ancestral role in bilaterian anterior‐posterior axis patterning

**DOI:** 10.1111/ede.12245

**Published:** 2017-12-15

**Authors:** Emanuel Redl, Maik Scherholz, Tim Wollesen, Christiane Todt, Andreas Wanninger

**Affiliations:** ^1^ Faculty of Life Sciences, Department of Integrative Zoology University of Vienna Vienna Austria; ^2^ The Natural History Collections University of Bergen University Museum Bergen Norway

## Abstract

The homeodomain transcription factors *six3* and *otx* are involved in patterning the anterior body and parts of the central nervous system (CNS) in bilaterians. Their similar expression patterns have been used as an argument for homology of heads, brains, segmentation, and ciliated larvae. We investigated the developmental expression of *six3* and *otx* in the aplacophoran mollusk *Wirenia argentea*. *Six3* is expressed in subepithelial cells delimiting the apical organ of the solenogaster pericalymma larva. *Otx* is expressed in cells of the prototroch and adjacent regions as well as in posterior extensions of the prototrochal expression domain. Advanced larvae also show pretrochal *otx* expression in the developing CNS. Comparative analysis of *six3* and *otx* expression in bilaterians argues for an ancestral function in anterior‐posterior body axis patterning but, due to its presence in animals lacking a head and/or a brain, not necessarily for the presence of these morphological structures in the last common ancestor (LCA) of bilaterians. Likewise, the hypothesis that the posterior border of *otx* expression corresponds to the border between the unsegmented head and the segmented trunk of the LCA of protostomes is not supported, since *otx* is extensively expressed in the trunk in *W. argentea* and numerous other protostomes.

## 1 INTRODUCTION

The homeodomain transcription factors *six3* and *otx* are involved in patterning the anterior body region and the central nervous system (CNS) in a variety of lophotrochozoans (Bely & Wray, 2001; Bruce & Shankland, 1998; Buresi, Baratte, Da Silva, & Bonnaud, 2012; Ogura et al., 2013), ecdysozoans (Browne, Schmid, Wimmer & Martindale, 2006; Finkelstein & Boncinelli, 1994; Hirth et al., 2003; Hunnekuhl & Akam, 2014; Janssen, Budd, & Damen, 2011; Li et al., 1996; Kawakami, Sato, Ozaki, & Ikeda, 2000; Martín‐Durán, Janssen, Wennberg, Budd, & Hejnol, 2012; Pechmann, McGregor, Schwager, Feitosa, & Damen, 2009), and deuterostomes (Cañestro, Bassham, & Postlethwait, 2005; Finkelstein & Boncinelli, 1994; Kawakami et al., 2000; Simeone et al., 1993). Thereby, their expression domains are arranged in an anterior to posterior sequence with *six3* being expressed slightly more anterior than *otx* (Eriksson, Samadi, & Schmid, 2013; Martín‐Durán, Vellutini, & Hejnol, 2015; Lowe et al., 2003; Oliver et al., 1995; Pani et al., 2012; Steinmetz et al., 2010). This identical spatial succession of expression domains has led to the conclusion that this pattern is ancestral for bilaterians (Steinmetz et al., 2010). Their function and/or spatial expression pattern, together with that of representatives of other gene families, such as homeobox and Pax genes, has even been used as an argument for homology of bilaterian heads and anterior centralized parts of the nervous system, that is, brains (Bruce & Shankland, 1998; Hirth et al., 2003; Hunnekuhl & Akam, 2014; Lichtneckert & Reichert, 2005; Reichert & Simeone, 2001), or the presence of segmentation in the last common ancestor (LCA) of protostomes (Steinmetz, Kostyuchenko, Fischer, & Arendt, 2011). Furthermore, *otx* is additionally expressed in more posterior parts of the nervous system in several representatives of the Bilateria (e.g., Bruce & Shankland, 1998; Browne et al., 2006; Eriksson et al., 2013; Hirth et al., 2003; Li et al., 1996; Pechmann et al., 2009).

In planktonic ciliated larvae of cnidarians, protostomes, and deuterostomes, s*ix3* is expressed anteriorly in the area around the major sensory structure of these larvae, the apical organ, but never in the cells that bear the cilia of the apical tuft (Hiebert & Maslakova, 2015ab; Marlow, Matus, & Martindale, 2013; Marlow et al., 2014; Poustka et al., 2007; Perry et al., 2015; Steinmetz et al., 2010; Santagata, Resh, Hejnol, Martindale, & Passamaneck, 2012; Sinigaglia, Busengdal, Leclère, Technau, & Rentzsch, 2013; Vöcking, Kourtesis, & Hausen, 2015; Vellutini, Martín‐Durán, & Hejnol, 2017; Wei, Yaguchi, Yaguchi, Angerer, & Angerer, 2009). Only in *Platynereis dumerilii*, expression in a low number of other cells belonging to the apical organ has been demonstrated (Marlow et al., 2014). In nemertines and gastropods, *six3* is additionally expressed in the ciliary bands (Hiebert & Maslakova, 2015b; Perry et al., 2015) and in echinoids further expression domains around the abapical pole as well as in the ciliary bands are present (Poustka et al., 2007; Wei et al., 2009).

*Otx* is expressed more abapically than *six3*, in the region of the ciliary bands in both protostome and deuterostome planktonic larvae (Arenas‐Mena & Wong, 2007; Arendt, Technau, & Wittbrodt, 2001; Gan et al., 1995; Harada et al., 2000; Hinman, Nguyen, & Davidson, 2003; Lowe & Wray, 1997; Lowe, Issel‐Tarver, & Wray, 2002; Nederbragt, te Welscher, van den Driesche, van Loon, & Dictus, 2002; Perry et al., 2015; Shoguchi, Harada, Numakunai, & Satoh, 2000; Steinmetz, Zelada‐Gonzáles, Burgtorf, Wittbrodt, & Arendt, 2007; Steinmetz et al., 2011, 2010; Vöcking et al., 2015; Vellutini et al., 2017). Additionally, *otx* expression is present in parts of the posttrochal ectoderm and in the gut in larvae of annelids, gastropods, and echinoids (Arenas‐Mena & Wong, 2007; Boyle, Yamaguchi, & Seaver, 2014; Gan et al., 1995; Nederbragt et al., 2002; Perry et al., 2015) as well as in the CNS of annelid and gastropod larvae (Arendt et al., 2001; Boyle et al., 2014; Perry et al., 2015; Vöcking et al., 2015). In the cnidarian *Nematostella*, a part of the expression domain is in the ectoderm of the developing tentacles, which originate from the likewise strongly ciliated oral ectoderm (Mazza, Pang, Martindale & Finnerty, 2007).

The expression of *six3* around the apical organ and of *otx* more posteriorly in the region of the ciliary bands is part of an expression pattern which is widespread within eumetazoans and has been used as evidence for the presence of planktonic ciliated larvae with an apical organ and ciliary bands in the life cycle of the last common ancestor of the Bilateria or even the Eumetazoa (Arendt et al., 2001; Marlow et al., 2014). This is basically a modification of a hypothesis that was originally formulated on morphological grounds for all metazoans, that is, including the Porifera (Jägersten, 1972). Yet, similar “apical‐organ‐like” expression patterns have also been reported for the anterior pole of insect and myriapod embryos (Posnien, Koniszewski, Hein, & Bucher, 2011; Hunnekuhl & Akam, 2014).

**Figure 1:**
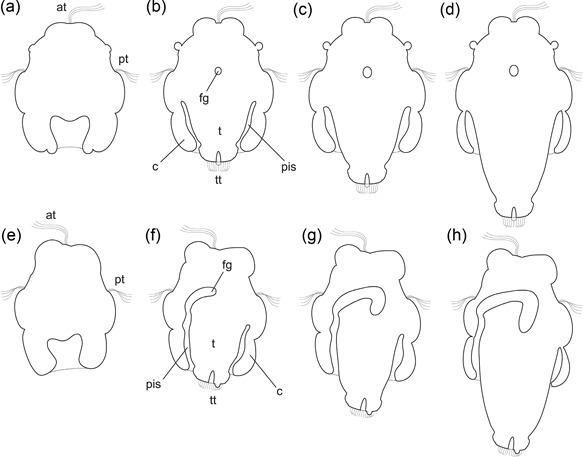
Schematic representations of larvae of *Wirenia argentea*. Apical is up in all panels; ventral is left in (e)–(h); (a)–(d) represent horizontal sections; (e)–(h) sagittal sections. (a) and (e) 0–1 days post hatching (dph) larva. (b) and (f) 6–7 dph larva. (c) and (g) 10–11 dph larva. (d) and (h): 14–15 dph larva. Abbreviations: at, apical tuft; c, calymma; fg, foregut; pis, peri‐imaginal space (*sensu* Salvini‐Plawen, 1980), that is, the cavity between the calymma and the outgrowing trunk of the animal; pt, prototroch; t, outgrowing trunk; tt, telotroch.

With regard to mollusks, larval expression patterns of these two genes are only known from polyplacophorans and gastropods, that is, from trochophore and veliger larvae, respectively (Nederbragt et al., 2002; Perry et al., 2015; Vöcking et al., 2015). Between those, profound differences were discovered, such as posttrochal *otx* expression in gastropod larvae and its absence in polyplacophorans, or the untypical expression of *six3* in the shell gland in *Crepidula*. We investigated developmental expression of *six3* and *otx* in another molluscan larval type, the pericalymma (or test cell) larva of an aplacophoran mollusk, the solenogaster *Wirenia argentea* Odhner (1921). This larva possesses the usual features of a trochophore‐type larva, such as an apical organ and a prototroch, but is additionally characterized by a special larval organ, the calymma (larval test, apical cap), which bears the prototroch, encloses the larval body for a considerable time during development, and is later shed or absorbed (see Figure 1 and, e.g., Todt & Wanninger, 2010). Comparative data from a third molluscan larval type will consequently provide more insight into ancestral and derived character states of *six3* and *otx* expression patterns in molluscan larval development.

## 2 MATERIALS AND METHODS

### 2.1 Animal cultures

Specimens of *Wirenia argentea* were collected from muddy bottoms in Hauglandsosen and Hjeltefjorden, both in the vicinity of Bergen, Norway, and were kept under laboratory conditions. The animals spawned fertilized eggs, which developed via free‐swimming larvae into metamorphosized juveniles, so that the entire developmental sequence could be followed. For a detailed account on collection, culture, and rearing see Redl, Scherholz, Todt, Wollesen, & Wanninger (2014) and Redl, Scherholz, Wollesen, Todt, & Wanninger (2016). The age of the larvae is given in days posthatching (dph), whereby 0–1 dph is used for larvae ranging from newly hatched to an age of 24 hr.

### 2.2 Transcriptome data, cloning, and probe synthesis

RNA extraction and sequencing of the transcriptome was done as described in Redl et al. (2016). The obtained paired‐end reads of an average read length of 100 bp were subsequently filtered (rRNA removal). After filtering, adapter sequences were trimmed and low‐quality sequences were removed. The remaining sequences were normalized and assembled de novo into contigs with the assembler Trinity (Grabherr et al., 2011). Two assembled transcriptomes were generated, one comprising 163,084 contigs and approximately 145 Mbp, the other comprising 217,838 contigs and approximately 173 Mbp. The assemblies were analyzed and mined for the *six3* and *otx* orthologs according to the description in Redl et al. (2016). Primers with the following sequences were designed for amplifying parts of the genes (in case of *six3*, the part between the primers spans the whole conserved region of the gene, in *otx* it includes a major part of it):

*six3*: forward: 5′‐GACAACTCGTATCCAATGTAACACTC‐3′ (annealing temperature: 60°C); reverse: 5′‐CAAGGACGCACTGTGAATATTGGTTG‐3′ (annealing temperature: 63°C); 948 nucleotides length;

*otx*: forward: 5′‐ ACTTGACGTACTGGAGAACCTCTTCCAC‐3′ (annealing temperature: 65.8°C); reverse: 5′‐ACAGTCTGTATTACTTCGGGACATGGTAT‐3′ (annealing temperature: 63.4°C); 625 nucleotides length.

Primers were synthesized by Invitrogen, Life Technologies (Glasgow, United Kingdom). The nucleotide sequences of the whole contigs as well as the amino acid sequences of the coding regions are available from the National Center for Biotechnology Information (NCBI; Bethesda, MD; www.ncbi.nlm.nhi.gov) nucleotide sequence database (Accession numbers: MG214871 (*six3*), MG214870 (*otx*)). Before uploading, the nucleotide sequences of the contigs were edited using sequence data from the cloned gene fragments.

First Strand cDNA synthesis, amplification and cloning of the gene fragments as well as probe synthesis were done as described in Redl et al. (2016) with the following modifications: For *six3*, only adult RNA was used for cDNA synthesis, the temperature range for the touchdown PCR was 64–59°C, and after the following gel electrophoresis, DNA was extracted using a silica‐based protocol after Boyle & Lew (1995), whereby four gel slice volumes of 6M sodium iodide and a suspension of silicon dioxide (Sigma‐Aldrich, St. Louis, MO) in water were used. For probe synthesis, 1 μl Protector RNase Inhibitor was added to the transcription reaction. In case of *otx*, only adult RNA was used for cDNA synthesis and the resulting cDNA was not diluted with water. The temperature range for the touchdown PCR was 63–57°C and the purification step of the amplified plasmid inserts immediately before probe synthesis was omitted. For probe synthesis, 0.5 μl Protector RNase Inhibitor (Roche, Basel, Switzerland) were added to the transcription reaction and the T7 RNA polymerase with the corresponding transcription buffer was used.

### 2.3 In situ hybridization

In situ hybridization experiments were conducted following the protocol described in Redl et al. (2016) with the following modifications: For *six3*, prehybridization and hybridization temperature was 58–60°C and the colorimetric reaction took 1‐20 hr. For *otx*, the prehybridization and hybridization temperature was 58–63°C and the color was developed for 2.5–6.5 hr.

### 2.4 Clearing and mounting

Clearing was done at room temperature (RT). Specimens were stepped into deionized water and washed therein four times for 5 min each, then stepped into 100% ethanol, washed three times for 5 min each therein, transferred into a mixture of benzyl benzoate and benzyl alcohol in a ratio of 1:1 or 2:1, and mounted in the respective mixture on microscope slides.

Uncleared specimens were mounted in glycerol, Fluoromount‐G (SouthernBiotech, Birmingham, AL), or 1× Roti‐Stock phosphate buffered saline (pH = 7.4; Carl Roth, Karlsruhe, Germany) on microscope slides.

### 2.5 Microscopy, 3D rendering, and image processing

The slides were analyzed and light micrographs were taken using either a Nikon SMZ25 stereo microscope equipped with a Nikon Digital Sight DS‐Ri1 camera and the software NIS‐Elements BR, Version 4.30.02 64bit (Nikon Corporation, Shinagawa, Tokyo, Japan), or an Olympus BX53 microscope equipped with an Olympus DP73 camera and the software cellSens Standard, Version 1.11 (Olympus Corporation, Shinjuku, Tokyo, Japan). Confocal microscopy was conducted using a Leica TCS SP5 II confocal laser scanning microscope equipped with the software Leica Application Suite Advanced Fluorescence (LAS AF), Version 2.6.0 to 2.6.3 (Leica Microsystems, Wetzlar, Germany) as described in Redl et al. (2016). 3D rendering and processing of the confocal image stacks was done with Imaris x64, Version 7.3.1 (Bitplane, Zurich, Switzerland). The autofluorescence signal of the larvae was imaged as volume rendering in blend mode (light blue in figures) and the gene expression signal as surface rendering (yellow in figures). Final image processing and generation of the schematic drawings was done with Adobe Photoshop CS5 Extended, Version 12.0 to 12.0.4 × 64, Adobe Photoshop CS6 Extended, Version 13.0.1 × 64, Adobe Photoshop CC 2015 to 2017, Adobe Illustrator CS5, Version 15.0.0, and Adobe Illustrator CC 2015 and 2017 (Adobe Systems, San José, CA).

For *six3*, approximately 70 specimens were processed and investigated, of which 20 were scanned. In case of *otx*, approximately 140 specimens were processed and investigated and 15 of them were scanned.

### 2.6 Bioinformatics analysis of the *six3* ortholog of *Wirenia argentea*

Gene orthology of *six3* was determined by phylogenetic reconstruction. A FASTA‐formatted file was generated with the identified correct ORF of the inferred amino acid sequence for the cloned gene and representative homologs from other metazoan taxa taken from the NCBI databases (see Supplementary Figure S1). Sequence alignment was performed with the online version of MAFFT (http://www.ebi.ac.uk/Tools/msa/mafft/; Katoh & Standley, 2013) with the following modifications to the standard setting: MAXITERATE à 100 (long run); PERFORM FFTS à localpair. In order to remove non‐conserved regions, the resultant alignment was trimmed by eye using BioEdit (Hall, 1999). The phylogenetic analysis was carried out with the program MrBayes v.3.2.6 (Ronquist et al., 2012) using a specified evolutionary model determined by applying the Akaike Information Criterion (AIC), as implemented in ProtTest 3 (Darriba, Taboada, Doallo, & Posada, 2011). MrBayes was operated using the following parameters: evolutionary model JTT + G; 30,000,000 generations; sample frequency 1,000; burn‐in 7,500,000. After the removal of 25% of the sampled trees as burn‐in, the final phylogenetic tree was created with FigTree v1.4.2 (http://tree.bio.ed.ac.uk/software/figtree/; Rambaut, 2014), and the generated tree was edited and enhanced with Adobe Illustrator CC 2015.

### 2.7 Bioinformatics analysis of the *otx* ortholog of *Wirenia argentea*

Several otx and otp amino acid sequences were downloaded from the NCBI databases (see Supplementary Figure S3). All sequences were aligned with the inferred amino acid sequence of the contig from the assembled transcriptome, which was used to design the primers for the *otx* probe, using the ClustalW algorithm (cost matrix: BLOSUM; gap open cost: 10; gap extend cost: 0.1). The alignment was manually trimmed to a region that encompasses the conserved homeodomain and overlaps with the cloned fragment. A consensus tree was then calculated from this alignment using the neighbor‐joining (genetic distance model: Jukes‐Cantor) and the bootstrap algorithm (number of replicates: 10,000; support threshold: 50%). The tree was rooted using the otp cluster as outgroup. The bioinformatics analysis was performed with Geneious, Version 6.1.6 (Biomatters, Auckland, New Zealand). The graphics were edited with Adobe Photoshop CC 2015.

## 3 RESULTS

### 3.1 Gene orthology assessment

For *six3*, the multiple sequence alignment includes metazoan orthologs of all Six subfamilies and comprises the complete conserved Six‐domain and homeodomain as well as the 5′ and 3′ flanking regions (Supplementary Figure S1). Our transcriptomic data reveal the presence of both conserved domains in the six3 amino acid sequence of *Wirenia argentea*. The *six3* sequence of *W*. *argentea* can be unambiguously identified by using the BLAST algorithm and metazoan orthologs as well as by phylogenetic analysis of their predicted amino acid sequences. The Bayesian analysis of Six proteins includes all Six protein subfamilies (six1/2, six3/6, and six4/5) and demonstrates that our solenogaster six3 amino acid sequence clusters with its corresponding metazoan orthologs (Supplementary Figure S2).

For *otx*, the alignment demonstrates the presence of the homeodomain characteristic for otx in the inferred partial amino acid sequence of the contig from the assembled transcriptome, which was used to design the primers for the *otx* probe, clearly showing that the latter is part of the *otx* ortholog of *Wirenia argentea* (Supplementary Figure S3; cf. Buresi et al., 2012). Furthermore, the tree shows two distinctive clusters, one for otp and one for otx, substantiating this result (Supplementary Figure S4).

### 3.2 *Six3* expression

**Figure 2:**
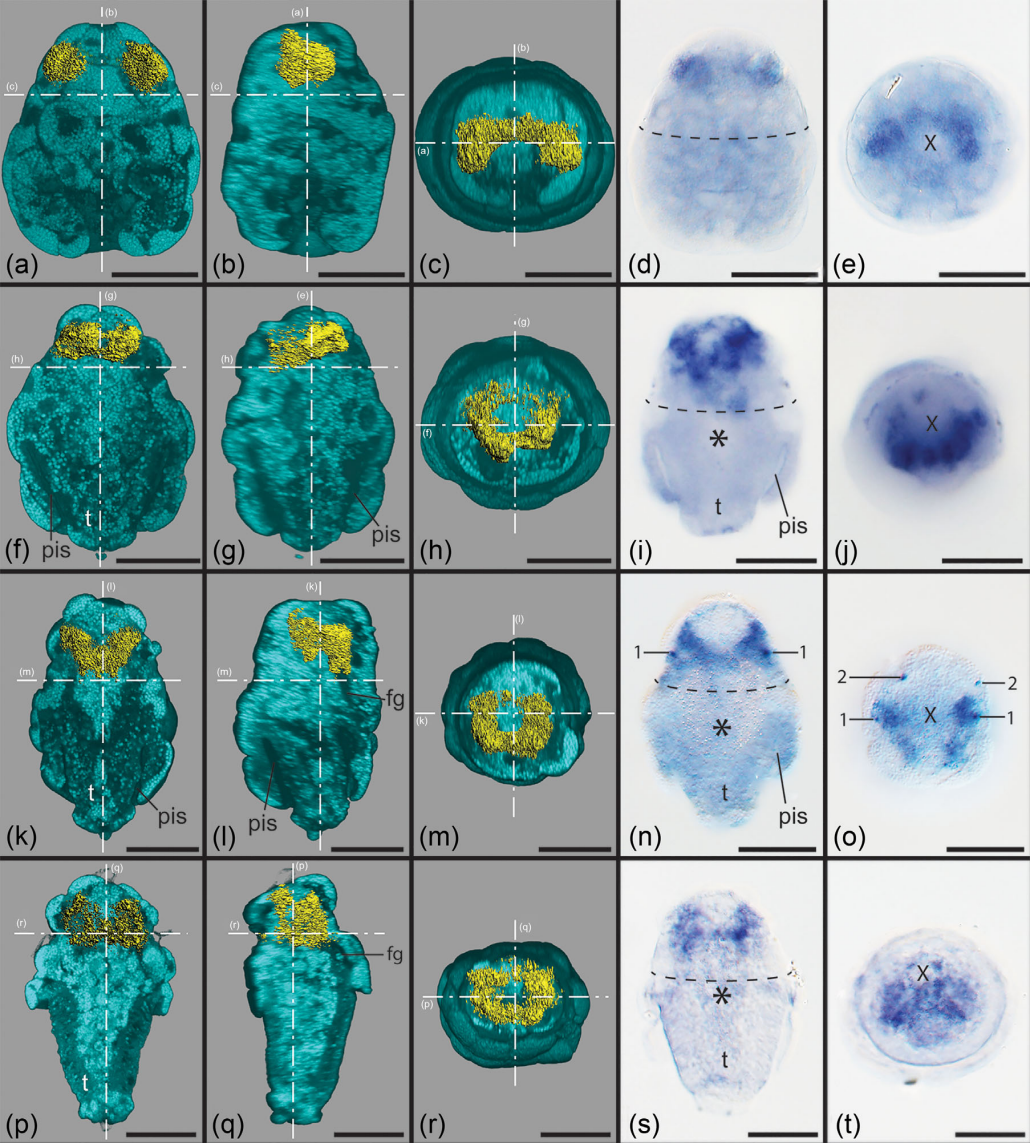
Expression of *six3* in larvae of *Wirenia argentea*. Apical/anterior is up in (a), (b), (d), (f), (g), (i), (k), (l), (n), (p), (q), and (s); dorsal is up in (c), (e), (h), (j), (m), (o), (r), and (t); scale bars equal 50 μm in all panels. (a)–(c), (f)–(h), (k)–(m), and (p)–(r) show 3D reconstructions of confocal scans with autofluorescence (light blue) and gene expression signal (yellow). The dashed and dotted lines labeled with capital letters show the section plane of the respective panel. (d), (e), (i), (j), (n), (o), (s), and (t) are light micrographs. The dashed line in (d), (i), (n), and (s) demarcates the region of the prototroch; the asterisk in (i), (n), and (s) demarcates the mouth opening; the X in (e), (j), (o), and (t) marks the region of the apical tuft. Panels (a)–(e), (f)–(h), (i)–(j), (k)–(m), (n)–(o), (p)–(r), and (s)–(t) show identical specimens in each case. (a) 0–1 days post hatching (dph) larva in ventral view with ventral part of autofluorescence signal omitted. (b) Same reconstruction as in (a) in right lateral view with right part of autofluorescence signal omitted. (c) Same reconstruction as in (a) and (b) in apical view with apical part of autofluorescence signal omitted. (d) and (e) 0–1 dph larva in ventral (d) and apical (e) view. (f) 6–7 dph larva in ventral view with ventral part of autofluorescence signal omitted. (g) Same reconstruction as in (f) in right lateral view with right part of autofluorescence signal omitted. (h) Same reconstruction as in (f) and (g) in apical view with apical part of autofluorescence signal omitted. (i) and (j) 6–7 dph larva in ventral (i) and apical (j) view. (k) 10–11 dph larva in ventral view with ventral part of autofluorescence signal omitted. (l) Same reconstruction as in (k) in right lateral view with right part of autofluorescence signal omitted. (m) Same reconstruction as in (k) and (l) in apical view with apical part of autofluorescence signal omitted. (n) and (o) 10–11 dph larva in ventral (n) and apical (o) view; 1 and 2 mark the additional pairs of *six3‐*expressing cells. (p) 14–15 dph larva in ventral view with ventral part of autofluorescence signal omitted. (q) Same reconstruction as in (p) in right lateral view with right part of autofluorescence signal omitted. (r) Same reconstruction as in (p) and (q) in apical view with apical part of autofluorescence signal omitted. (s) and (t) 14–15 dph larva in ventral (s) and apical (t) view. Abbreviations: fg, foregut, pis, peri‐imaginal space (*sensu* Salvini‐Plawen, 1980), that is, the cavity between the calymma and the outgrowing trunk of the animal; t, outgrowing trunk

**Figure 3:**
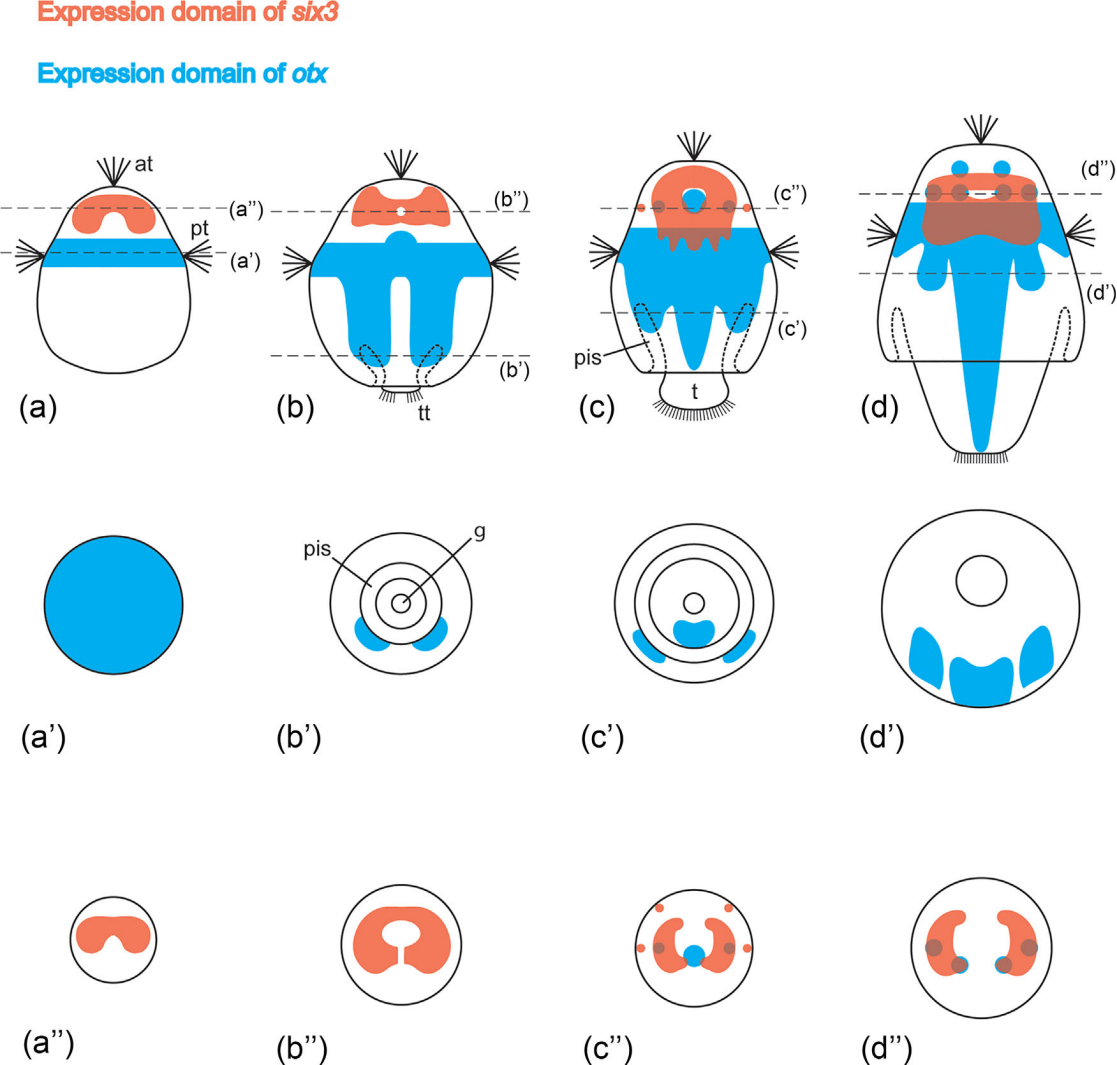
Schematic summary of *otx* and *six3* expression in larvae of *Wirenia argentea*. Apical/anterior is up in (a)–(d); dorsal is up in (a’)–(d’) and (a”)–(d”). In stages where expression was not fully consistent, the maximum extent of the expression domain is depicted. (a)–(d): Dorsal views of newly hatched (a), early (b), advanced (c), and late larvae (d). (a’)–(d’) and (a”)–(d”) Cross sections of corresponding stages at the position of the dashed lines in (a)–(d). Abbreviations: at, apical tuft; pis, peri‐imaginal space (*sensu* Salvini‐Plawen, 1980), that is, the cavity between the calymma and the outgrowing trunk of the animal; g, gut; pt, prototroch; t, outgrowing trunk; tt, telotroch

In newly hatched larvae, the most intense signal for *six3* expression was found in two comparatively large subepithelial cells lateral and adjacent to the cells of the apical organ (Figures 2a, 2b, and 2d). At their dorsal side, subepithelial cells that show a weaker s*ix3* expression signal are located between them, thus forming a connection between the main parts of the expression domain (Figures 2c, 2e, and 3a, 3a”). All s*ix3‐*expressing cells are covered by non‐expressing test cells and are not part of the apical organ. Later, the *six3* expression domain forms a ring encompassing apically positioned cells underneath the test cells, surrounding the cells of the apical organ proper (Figures 2f‐j, 3b and 3b”). The dorsal part of the ring shows a less intense expression signal and on its ventral side a small region that lacks expression entirely (Figures 2f, 2h, 2j, and 3b, 3b”). Advanced larvae still possess this ring‐like expression domain, but the ventral expression‐free region has disappeared and the ring is tilted insofar that its ventral part lies more posteriorly and the dorsal part more anteriorly. The signal intensity is still higher in the ventral and lateral regions (Figures 2k‐o, and 3c, 3c”). Some individuals showed two additional pairs of s*ix3‐*expressing cells, one laterally and one more dorsally situated (Figures 2n, 2o and 3c, 3c”). Late larvae show a similar expression domain but with a generally lower level of expression. No additional cells with *six3* expression were observed (Figures 2p‐t and 3d, 3d”).

### 3.3 *Otx* expression

**Figure 4:**
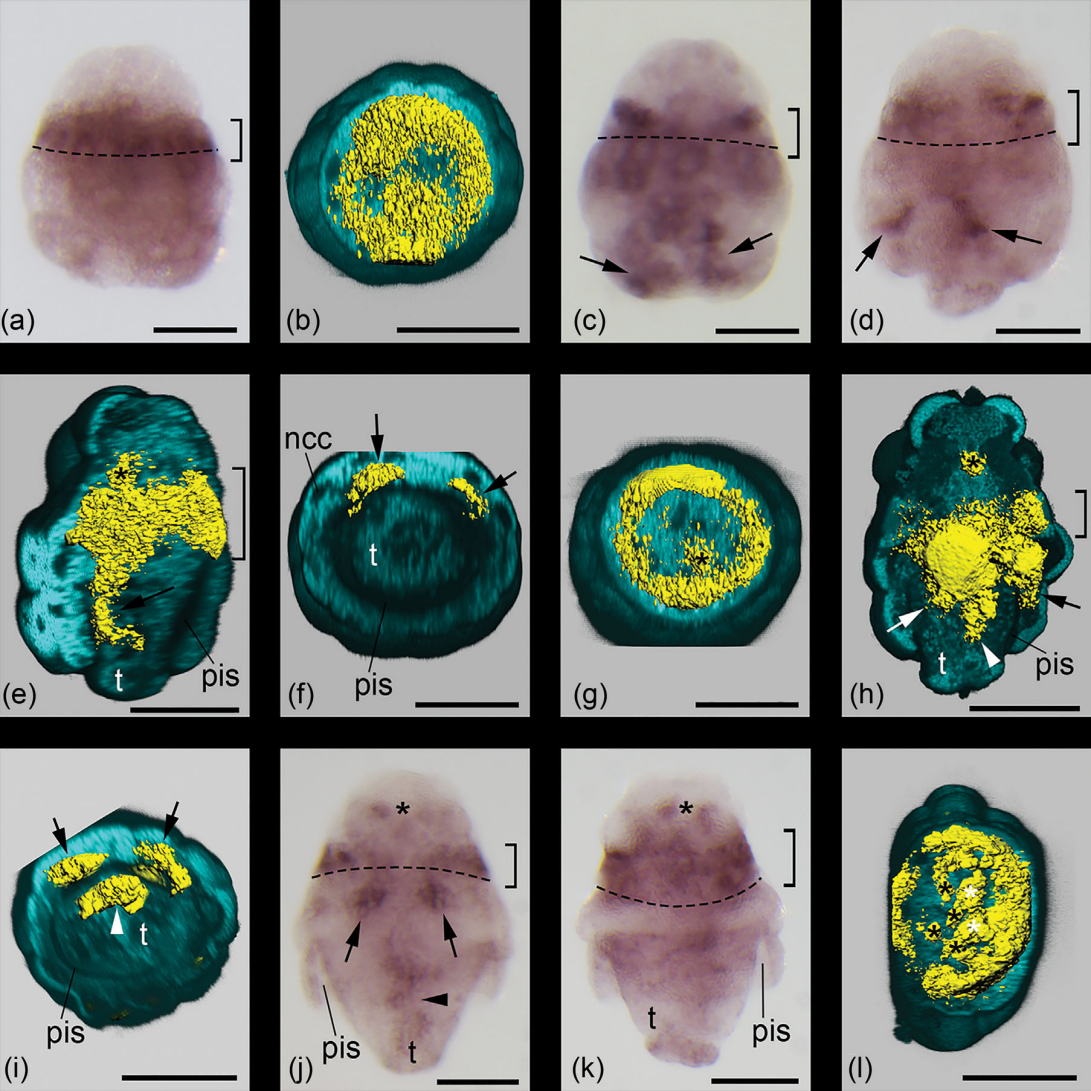
Expression of *otx* in larvae of *Wirenia argentea*. Apical/anterior is up in (a), (c), (d), (f), (h), and (k); dorsal is up in (b) and (e); ventral is up in (g) and (i), and right in (l); scale bars equal 50 μm in all panels. (a), (c), (d), (j), and (k) are light micrographs. The dashed line demarcates the region of the prototroch. (b), (e)–(i), and (l) show 3D reconstructions of confocal scans with autofluorescence (light blue) and gene expression signal (yellow). (a) 0–1 days post hatching (dph) larva showing prototrochal expression domain (bracket). (b) 0–1 dph larva in apical view with apical part of autofluorescence signal omitted showing the disk‐like shape of the prototrochal expression domain. (c) 2–3 dph larva showing prototrochal expression domain (bracket) and its abapical extensions (arrows). (d) 6–7 dph larva showing prototrochal expression domain (bracket) and its abapical extensions (arrows). (e) 6–7 dph larva in left ventrolateral view with left part of autofluorescence signal omitted showing the prototrochal expression domain (bracket), its left posttrochal extension (arrow), and the central pretrochal expression domain (asterisk). (f) Same specimen and reconstruction as in (e) in abapical view with abapical part of autofluorescence signal omitted to reveal both posttrochal extensions of the prototrochal expression domain; note that the expression is restricted to the proximal side of the calymma, that is, to the prospective ectoderm. (g) Same specimen and reconstruction as in (e) and (f) in apical view with apical part of autofluorescence signal omitted to reveal the central pretrochal expression domain (asterisk). (h) 10–11 dph larva in right ventrolateral view with ventral part of autofluorescence signal omitted showing the prototrochal expression domain (bracket), its paired (arrows) and unpaired posttrochal extension (arrowhead), and the central pretrochal expression domain (asterisk). (i) Same specimen and reconstruction as in (h) in abapical view with abapical part of autofluorescence signal omitted to reveal the paired (arrows) as well as the unpaired posttrochal extension (arrowhead) of the prototrochal expression domain; note that in the paired extensions the expression is restricted to the proximal side of the calymma, that is, to the prospective ectoderm, while the unpaired extension is situated in the trunk. (j) 14–15 dph larva showing the prototrochal expression domain (bracket), its paired (arrows) and unpaired posttrochal extension (arrowhead), and the pretrochal expression (asterisk). (k) 14–15 dph larva showing the prototrochal expression domain (bracket) and the pretrochal expression (asterisk). (l) Same specimen as in (j) in apical view with apical part of autofluorescence signal omitted to reveal the pretrochal expression domain (black asterisks) and the paired expression domain (white asterisks) on the level of the ring‐like prototrochal expression domain. Abbreviations: ncc, nucleus of calymma cell; pis, peri‐imaginal space (*sensu* Salvini‐Plawen, 1980), that is, the cavity between the calymma and the outgrowing trunk of the animal; t, outgrowing trunk

Throughout development *otx* is expressed in the prototroch and adjacent regions, whereby initially approximately two thirds of the expression domain are situated anteriorly and one third is situated posteriorly of the prototroch. *Otx* expression is purely confined to this region in newly hatched larvae (Figures 3a and 4a). 3D reconstructions show that, at this stage, the expression domain is more or less disk‐shaped, extending far into the central regions of the animal (Figures 3a’ and 4b). Shortly afterwards, *otx* expression is also present in the posttrochal part of the larvae in the form of a pair of abapical extensions of the prototrochal expression domain, which are situated in the inner cell layer of the calymma, that is, in the ectoderm (Figures 3b, 3b’ and 4c–f). The expression in the prototrochal region has now become more confined to the outermost cells and is thus more ring‐like, while in the central region a more or less spherical expression domain is present, which is slightly shifted toward the pretrochal part of the larva (Figures 3b and 4g). In later stages this part of the expression pattern occupies a distinct pretrochal position and is sometimes flanked by a pair of smaller spherical domains with *otx* expression (Figures 3c and 4h). The paired extensions of the prototrochal expression domain have shortened but still reach as far as to the parts of the calymma that surround the peri‐imaginal space. Additionally, a third, unpaired posterior extension of the prototrochal expression domain has developed (Figures 3c and 4h). In contrast to the paired extensions, it is situated on the ventral side of the outgrowing trunk and not in the calymma (Figures 3c’ and 4i). Compared to earlier stages, the ring‐like prototrochal expression has shifted toward the apical side. During subsequent development, the prototrochal expression domain changes insofar, as it is almost entirely confined to the pretrochal side. The paired extensions of the prototrochal expression domain shorten further so that they do not reach the peri‐imaginal space anymore, while the unpaired extension elongates together with the outgrowing trunk (Figures 3d, 3d’ and 4j). Furthermore, the pretrochal expression now consists of a higher number (six to eight) of spherical expression domains and a paired expression domain is found on the ventral side of the animal in the area surrounded by the ring‐like prototrochal expression (Figures 3d and 4k‐l). In juvenile animals, no *otx* expression was found (data not shown).

## 4 DISCUSSION

### 4.1 *Six3* and *otx* in bilaterian anterior‐posterior patterning

The expression patterns of *six3* and *otx* in the larva of *Wirenia argentea* show a clear anterior to posterior sequence with a region of overlap in advanced to late larvae, which is consistent with the situation in other bilaterians (Eriksson et al., 2013; Hunnekuhl & Akam, 2014; Lowe et al., 2003; Martín‐Durán et al., 2015; Pani et al., 2012; Oliver et al., 1995; Steinmetz et al., 2010; Vöcking et al., 2015). This consistency of the spatial expression sequence and its independence of the mode of development (direct vs. indirect) strongly hints toward an ancestral role of these two genes in bilaterian anterior‐posterior body axis patterning (see also Lowe et al., 2003; Steinmetz et al., 2010). This does not necessarily imply that the LCA of bilaterians possessed a distinct head and a brain as previously proposed (Bruce & Shankland, 1998; Hirth et al., 2003; Hunnekuhl & Akam, 2014; Lichtneckert & Reichert, 2005; Reichert & Simeone, 2001) because the topography of the expression domains is conserved even in representatives without a distinct head, such as solenogasters (this study) or nemertines (Martín‐Durán et al., 2015), as well as in animals with a largely diffuse nervous system, such as enteropneusts (Lowe et al., 2003). Rather, the ancestral role of *six3* and *otx* was probably in patterning the anterior‐posterior body axis (which may have included longitudinal nerve cords). With the evolution of heads and brains in the diverse lineages both genes were co‐opted to pattern also the parts of these novel anterior structures. As a consequence, neither the notion can be upheld that the posterior border of *otx* expression can be homologized throughout the protostomes as the border between an unsegmented “head” and a segmented trunk of the LCA of protostomes (Steinmetz et al., 2011). In lophotrochozoans, developmental *otx* expression in the trunk is present in solenogasters (this study), gastropods (Nederbragt et al., 2002; Perry et al., 2015), and several annelids (Arenas‐Mena & Wong, 2007; Boyle et al., 2014; Bruce & Shankland, 1998). In ecdysozoans, it has been shown in insects, crustaceans, and myriapods (Browne et al., 2006; Hirth et al., 2003; Janssen et al., 2011; Li et al., 1996). The lack of posttrochal *otx* expression in *Platynereis dumerilii* (Arendt et al., 2001; Steinmetz et al., 2011, 2007) is probably due to secondary loss (see also section 4.2).

### 4.2 *Six3* and *otx* in eumetazoan ciliated larvae

*Six3* expression in the apical region of *Wirenia argentea* is consistent with expression patterns of the gene in polyplacophoran and gastropod larvae (Perry et al., 2015; Vöcking et al., 2015). However, additional expression in the ciliary bands or the shell gland, as described from a gastropod veliger (Perry et al., 2015), seems to be lacking in aculiferan mollusks. The apical expression pattern is also in accordance with the pattern described in ciliated larvae of other lophotrochozoans (Hiebert & Maslakova, 2015ab; Marlow et al., 2014; Santagata et al., 2012; Steinmetz et al., 2010; Vellutini et al., 2017), as well as deuterostomes (Poustka et al., 2007; Wei et al., 2009) and cnidarians (Marlow et al., 2013; Sinigaglia et al., 2013), where it is also mainly expressed in the area around the apical organ but never in the apical tuft cells proper. As in gastropods, additional *six3* expression in the ciliary bands is present in nemertine pilidium and sea urchin pluteus larvae (Hiebert & Maslakova, 2015b; Wei et al., 2009). In the latter, as well as in the trilobed larvae of brachiopods, it is also expressed in the developing foregut (Poustka et al., 2007; Santagata et al., 2012). *Six3* expression at the abapical pole described from early developmental stages of echinoids seems to be unique to this group (Poustka et al., 2007; Wei et al., 2009). This distribution of expression patterns argues for a conserved *six3* expression in the apical region of eumetazoans, whereas expression in other parts of the body, such as ciliary bands or the gut, appear to be the effect of independent co‐option events into additional roles in the respective taxa.

Admittedly, the consistent expression in cells encircling the apical organ in eumetazoan larvae, including the pericalymma larva of *W. argentea*, is a tempting argument in favor of the presence of an anterior/apical sensory structure in the LCA of eumetazoans. Yet, the proposed homology of all ciliated larvae of eumetazoans (Marlow et al., 2014) appears too far flung. Besides the profound morphological disparity of eumetazoan larval types, anterior cell populations with an “apical organ‐like” gene expression profile are also present in embryos of insects and myriapods (Hunnekuhl & Akam, 2014; Posnien et al., 2011) and *six3* expression at the anterior pole seems to be a general ancestral feature for bilaterians (see section 4.1). Furthermore, differences in the molecular machinery that builds the apical organs in protostomes and deuterostomes do exist, such as, for example, the transcription factors *NK2.1* and *HNF6* that play a role in apical tuft formation in echinoderms but not in gastropods (Dunn et al., 2007).

The expression of *otx* in the prototroch and adjacent regions of the larva of *W. argentea* is consistent with data on polyplacophorans and gastropods. Expression in the developing CNS is also present in polyplacophorans and in the caenogastropod *Crepidula* but doubtful in *Patella*, a member of the patellogastropods, the putative sister‐group to all other extant gastropods (but see Zapata et al., 2014, 2015). Posttrochal ectodermal expression of *otx* is restricted to the photoreceptors in polyplacophoran larvae, while in gastropod larvae it is distinctive and broad as in solenogasters (Nederbragt et al., 2002; Perry et al., 2015; Vöcking et al., 2015). Due to the fact that a broad posttrochal or posterior expression of *otx* as well as an expression in the developing CNS is also present in annelid larvae (Arendt et al., 2001; Arenas‐Mena & Wong, 2007; Boyle et al., 2014), we consider its putative lack of expression in the CNS of *Patella* larvae and the restricted posttrochal expression of *otx* in polyplacophoran larvae as derived character states. Interestingly, however, the posttrochal expression seems to be also lacking in larvae of the model polychaete annelid *Platynereis dumerilii* (Arendt et al., 2001; Steinmetz et al., 2011, 2007), probably another case of secondary loss (see section 4.1).

Prototrochal expression of *otx* is widespread among annelid larvae (Arenas‐Mena & Wong, 2007; Arendt et al., 2001; Steinmetz et al., 2011, 2007; for an exception, see the polychaete *Capitella telata*, Boyle et al., 2014). It may thus have been present in the LCA of mollusks and annelids, but the expression of *otx* in the ciliary band of a bryozoan larva (Vellutini et al., 2017) could hint to an even earlier evolutionary emergence of this character within the lophotrochozoans.

In deuterostome larvae, *otx* is often expressed in ciliary bands although it is lacking in several groups of echinoderms (Gan et al., 1995; Harada et al., 2000; Hinman et al., 2003; Lowe & Wray, 1997; Lowe et al., 2002; Shoguchi et al., 2000). Due to this rather patchy occurrence we do not consider the expression pattern of *otx* as a good argument for a homology of ciliated larvae of protostomes and deuterostomes. As a matter of fact, ciliary bands are highly innervated structures throughout the bilaterians (see, e.g., Hay‐Schmidt, 2000; Wanninger, 2009) and *otx* expression could simply reflect the presence of neurons and not constitute a phylogenetic signal (Dunn et al., 2007). This finds some support in the fact that several parts of the *otx*‐expression pattern we found in solenogasters seem to be related to the development of the nervous system. In the inner part of the episphere they probably mark cells of the developing centralized anterior nervous system, and the ventral expression in the trunk is probably linked to the development of the ventral nervous system as it is the case in annelids and insects (Boyle et al., 2014; Bruce & Shankland, 1998; Hirth et al., 2003; Li et al., 1996). In solenogaster larvae, a ciliated structure, the developing foot sole, is situated in this position and is underlain by a dense nerve plexus (Redl et al., 2014; Todt & Wanninger, 2010). The expression on the inner side of the calymma might be connected with the development of the lateral nerve cords, since these regions represent prospective trunk ectoderm (cf. Todt & Wanninger, 2010).

When it comes to the comparison with cnidarian larvae, even more severe difficulties arise concerning potential homology of larval forms. In planula larvae, the ectoderm of the developing tentacles, which originate from the ciliated oral ectoderm, expresses *otx*. It has thus been argued that the ciliated oral ectoderm of cnidarian larvae is homologous to the diverse ciliary bands in bilaterian larvae (Marlow et al., 2014; Mazza et al., 2007). In our opinion, this hypothesis is too speculative since it attempts to homologize morphologically different structures based on the expression pattern of a single gene, which is not even expressed in comparable developmental stages. Furthermore, in planula larvae of *Nematostella*, *otx* is expressed in the apical organ in cells of the apical tuft (Mazza et al., 2007), an expression pattern, which has never been found in any bilaterian.

## 5 CONCLUSIONS

The spatial expression sequence of *six3* and *otx* in bilaterian development supports an ancestral function in anterior‐posterior body axis patterning. Given that this expression signature is consistently present in morphologically highly diverse phyla, even in those whose representatives lack a distinct head or a centralized nervous system, it does not support hypotheses that argue for the presence of a head or a brain in the LCA of bilaterians. Rather, starting from its original function in regionalization of the anterior part of the body, an independent co‐option into patterning these novel structures in the different lineages is more likely. Likewise, due to the presence of extensive posttrochal expression of *otx* in *Wirenia argentea* and numerous other protostomes, the hypothesis that the posterior border of *otx* expression corresponds to the border between the head and the (segmented) trunk of the LCA of protostomes is not supported. Furthermore, we do not consider the expression patterns of *six3* and *otx* as good arguments for homology of eumetazoan ciliated larvae. Rather, the consistencies in the expression patterns of both genes among eumetazoan larvae can be explained by their ancestral role in patterning the anterior‐posterior body axis, which is also supported by their corresponding expression during direct development of insects and myriapods.

## Supporting information

Additional Supporting Information may be found online in the supporting information tab for this article.

**Figure S1**. Orthology assessment of *six3* of *Wirenia argentea* by alignment of amino acid sequences.

**Figure S2**. Orthology assessment of *six3* of *Wirenia argentea* by phylogenetic tree reconstruction.

**Figure S3**. Orthology assessment of *otx* of *Wirenia argentea* by alignment of amino acid sequences.

**Figure S4**. Orthology assessment of *otx* of *Wirenia argentea* by phylogenetic tree reconstruction.
