# Supplementary figures and images for: Expression of six3 and otx in Solenogastres (Mollusca) supports an ancestral role in bilaterian anterior‐posterior axis patterning
Source: Evol Dev. 2017 Dec 15;20(1):17–28. doi: 10.1111/ede.12245 (PMC5814893; doi:10.1111/ede.12245)

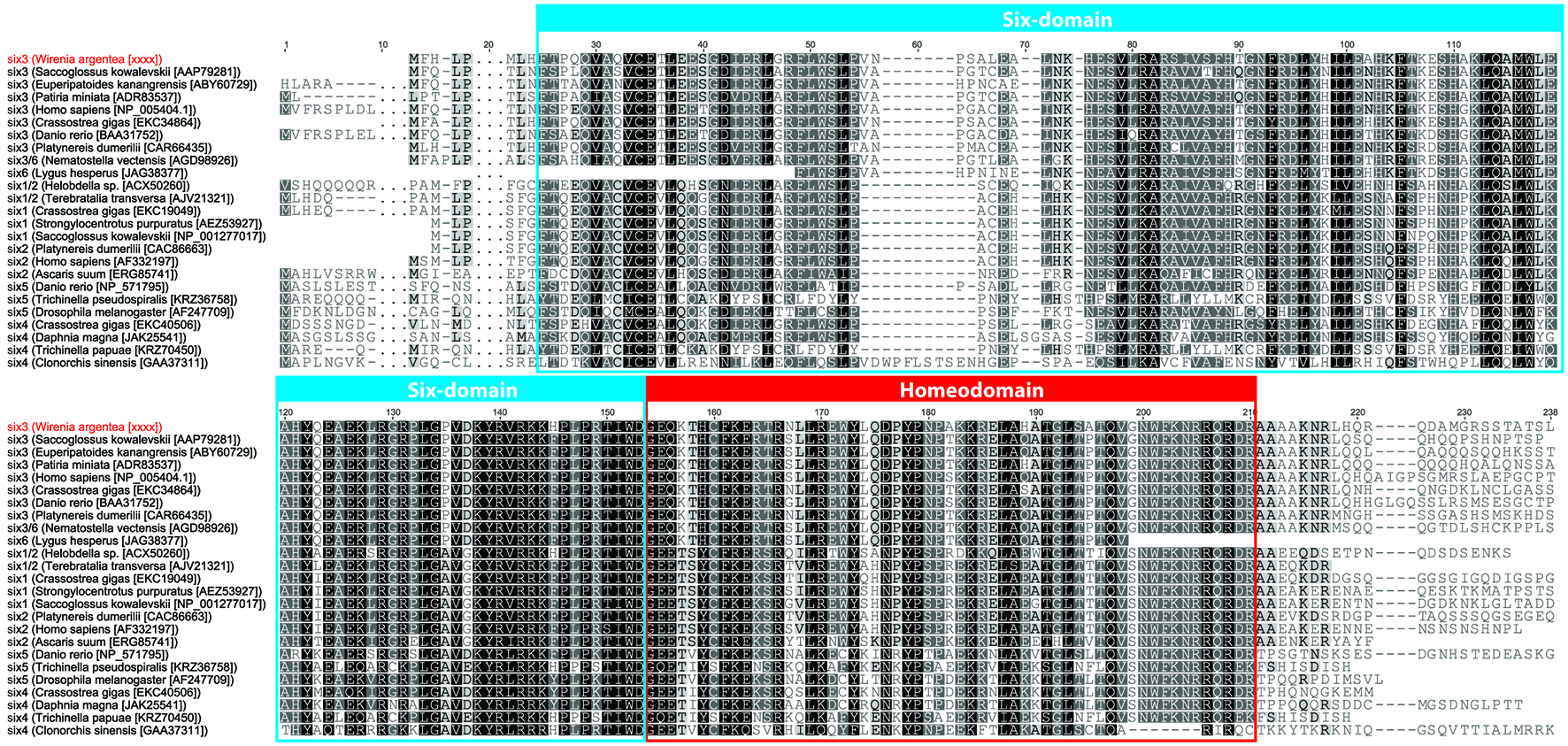

Supplement: Supplementary file 1 — Figure S1. Orthology assessment of six3 of Wirenia argentea by alignment of amino acid sequences. [file EDE-20-17-s001.tif]

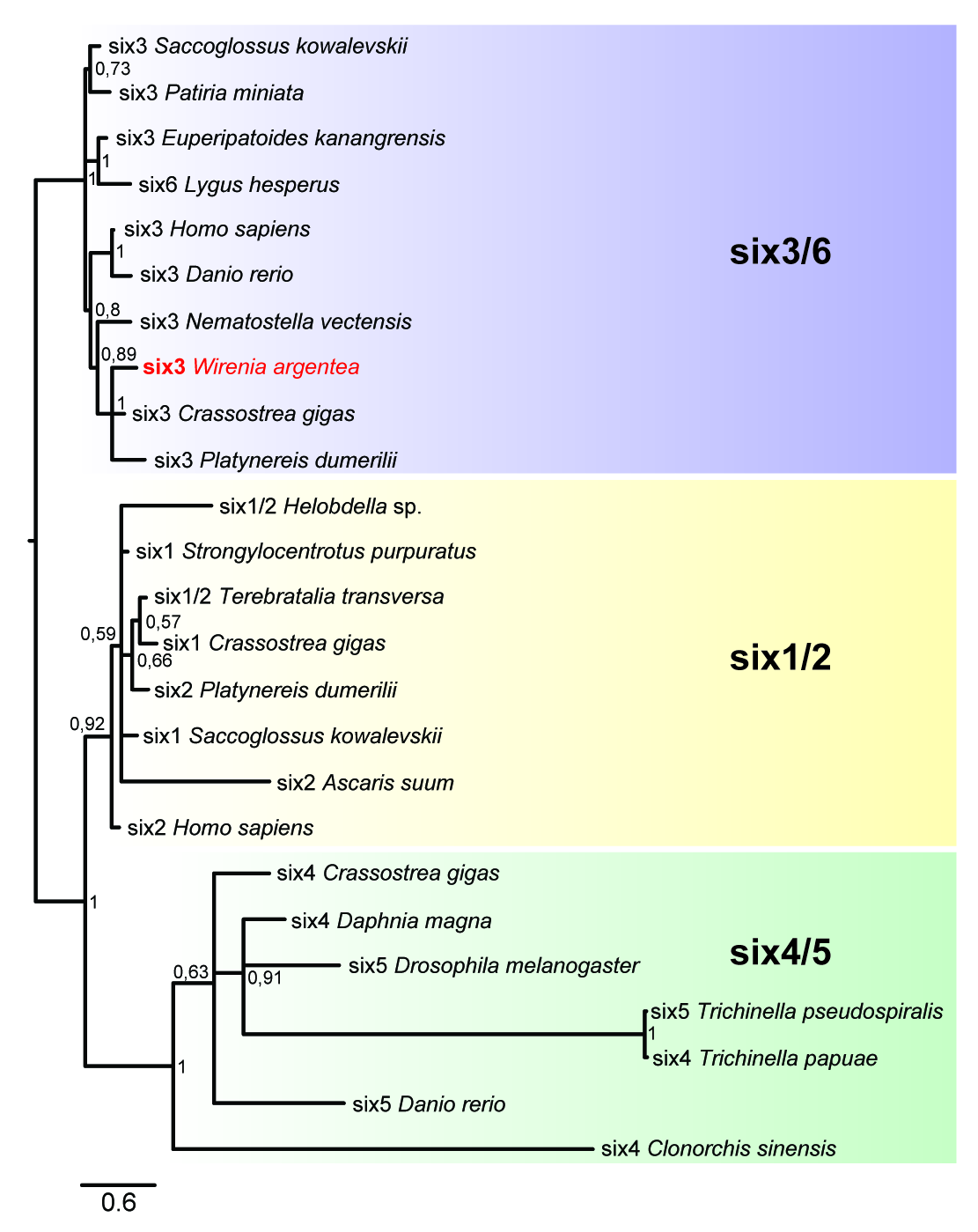

Supplement: Supplementary file 2 — Figure S2. Orthology assessment of six3 of Wirenia argentea by phylogenetic tree reconstruction. [file EDE-20-17-s002.tif]
